# Engineering Borophene Band Structure Through Thickness

**DOI:** 10.1002/advs.77716

**Published:** 2026-09-16

**Authors:** Matteo Jugovac, Marcin Szpytma, Tevfik Onur Menteş, Andrea Locatelli, Iulia Cojocariu

**Affiliations:** ^1^ Istituto di Struttura della Materia Consiglio Nazionale delle Ricerche Trieste Italy; ^2^ Dipartimento di Fisica Università degli Studi di Trieste Trieste Italy; ^3^ Faculty of Physics and Applied Computer Science AGH University of Kraków Krakow Poland; ^4^ Elettra Sincrotrone Trieste S.C.p.A. Trieste Italy

**Keywords:** borophene, electronic structure, hybridization, interface engineering, interfacial coupling, layer‐dependent properties

## Abstract

Borophene, the 2D allotrope of boron, exhibits electronic properties that strongly depend on the supporting substrate and film thickness. Understanding how interlayer and substrate interactions jointly regulate borophene's electronic properties remains a key challenge for the future exploitation of this emerging material. Here, monolayer and bilayer borophene are synthesized on W(110) to probe thickness‐induced changes in their band structure. In situ low‐energy electron microscopy and diffraction, combined with x‐ray photoelectron spectroscopy and angle‐resolved photoemission spectroscopy, reveal distinct borophene‐derived valence‐band fingerprints for the two thicknesses. While the monolayer adopts an ordered, epitaxial arrangement with substrate‐hybridized metallic states, the bilayer exhibits reduced visibility of these substrate‐hybridized features, long‐range order, and distinct valence‐band states. These results reveal a thickness‐induced electronic reorganization governed by structural reconstruction and interlayer interactions that accompany the formation of the second boron layer, which collectively shape the band structure of this emerging material.

## Graphical abstract

Borophene grown on W(110) shows a thickness‐dependent evolution of its structural and electronic properties. Surface microscopy, diffraction, XPS, and ARPES reveal distinct monolayer and bilayer phases. The formation of the second layer is accompanied by structural reconstruction, changes in chemical properties, and new ARPES features, demonstrating how dimensionality modifies borophene.

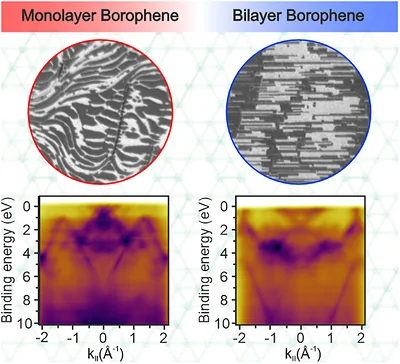

## 1 Introduction

Borophene has emerged as an elemental 2D metal with polymorphic lattices stabilized by periodic hexagonal vacancies within triangular boron networks, yielding anisotropic metallic transport and unconventional bonding motifs [1, 2, 3]. The first experimental syntheses on Ag(111) and Ag(110) demonstrated atomically thin sheets and nanoribbons of boron with long‐range order, while also confirming that phase selection and domain orientation are strongly governed by the symmetry of the supporting substrate [1, 2, 4]. Subsequent growth on Au(111), Cu(111), Ru(0001), and Ir(111) [5, 6, 7, 8, 9] expanded the accessible set of borophene polymorphs and established growth strategies capable of producing large structural domains on substrates with different coupling strengths. These advances consolidated borophene's position within the broader class of elemental 2D materials [10, 11, 12, 13, 14, 15, 16, 17, 18]. Reviews surveying synthesis‐structure‐property links emphasize a central theme: substrate choice and interfacial bonding are decisive for both structural order and electronic response [3, 19].

Electronic‐structure measurements and theoretical models have shown that several borophene polymorphs exhibit Dirac‐like dispersions [2, 20, 21]. On Ag(111), in situ scanning tunneling microscopy/spectroscopy (STM/STS) and angle‐resolved photoemission spectroscopy (ARPES) identified Dirac‐type features in β_12_‐like sheets, where periodic perturbations and substrate coupling split or reshape the cone‐like bands [20]. A complementary study of interfacial image‐potential states and work function has quantified electron transfer from metal substrates to borophene, testing and refining “self‐doping” models and further highlighting the dominant role of the interface in determining charge distribution [22]. On strongly interacting templates such as Ir(111), single‐phase monolayer borophene can be stabilized in a χ_6_ polymorph. However, electronic structure investigations of this interface demonstrated that the strong coupling at the borophene‐substrate interface hinders the observation of distinct borophene‐derived bands [23, 24].

Beyond the monolayer limit, layer thickness provides additional leverage for controlling electronic properties. Experimentally, bilayer borophene has been realized on Cu(111), where structural characterization combined with first‐principles analysis indicates strong interlayer interactions and direct bonding between the two boron sheets, yielding a phase that is structurally and electronically distinct from the monolayer [6]. Complementary theory has shown that the stability of bilayer borophene depends sensitively on the density of hexagonal holes and on the potential formation of interlayer B‐B bonds, rather than solely on van der Waals interlayer interactions [25]. Calculations on few‐layer borophene further predicted sizeable thickness‐induced changes in the band structure, including substantial band splitting while preserving metallicity. This highlights that thickness can strongly influence the electronic states even when the planar boron structure is retained [26]. Taken together, these studies suggest that bilayer borophene should not be viewed as a simple stacked analog of the monolayer, but as a distinct electronic phase in which interlayer bonding defines its structural and electronic properties. Recent first‐principles calculations for bilayer borophene on Ag(111), Au(111), and Al(111) predict that metal‐induced hybridization is concentrated primarily in the boron sheet directly contacting the metal, whereas the more remote sheet retains electronic features closer to those of the unsupported bilayer [27]. This layer‐selective picture provides a useful comparison for the experiment, although its transferability depends on the substrate, atomic registry, and bilayer structure.

Here, monolayer and bilayer borophene on W(110) are used as a model system to elucidate how dimensionality and interfacial coupling govern the electronic structure of 2D boron. In this respect, the W(110) surface provides a well‐defined template for controlling borophene growth and its interactions with the substrate. The structural evolution during preparation was monitored in situ by low‐energy electron diffraction (LEED), while low‐energy electron microscopy (LEEM) images were acquired at selected stages. X‐ray photoelectron spectroscopy (XPS) and angle‐resolved photoemission spectroscopy (ARPES) were employed to probe the chemical state of the synthesized phases and their band structure. This combined approach demonstrates that increasing the borophene thickness from one to two layers induces a pronounced reorganization of the electronic structure.

## 2 Results and Discussion

**FIGURE 1:**
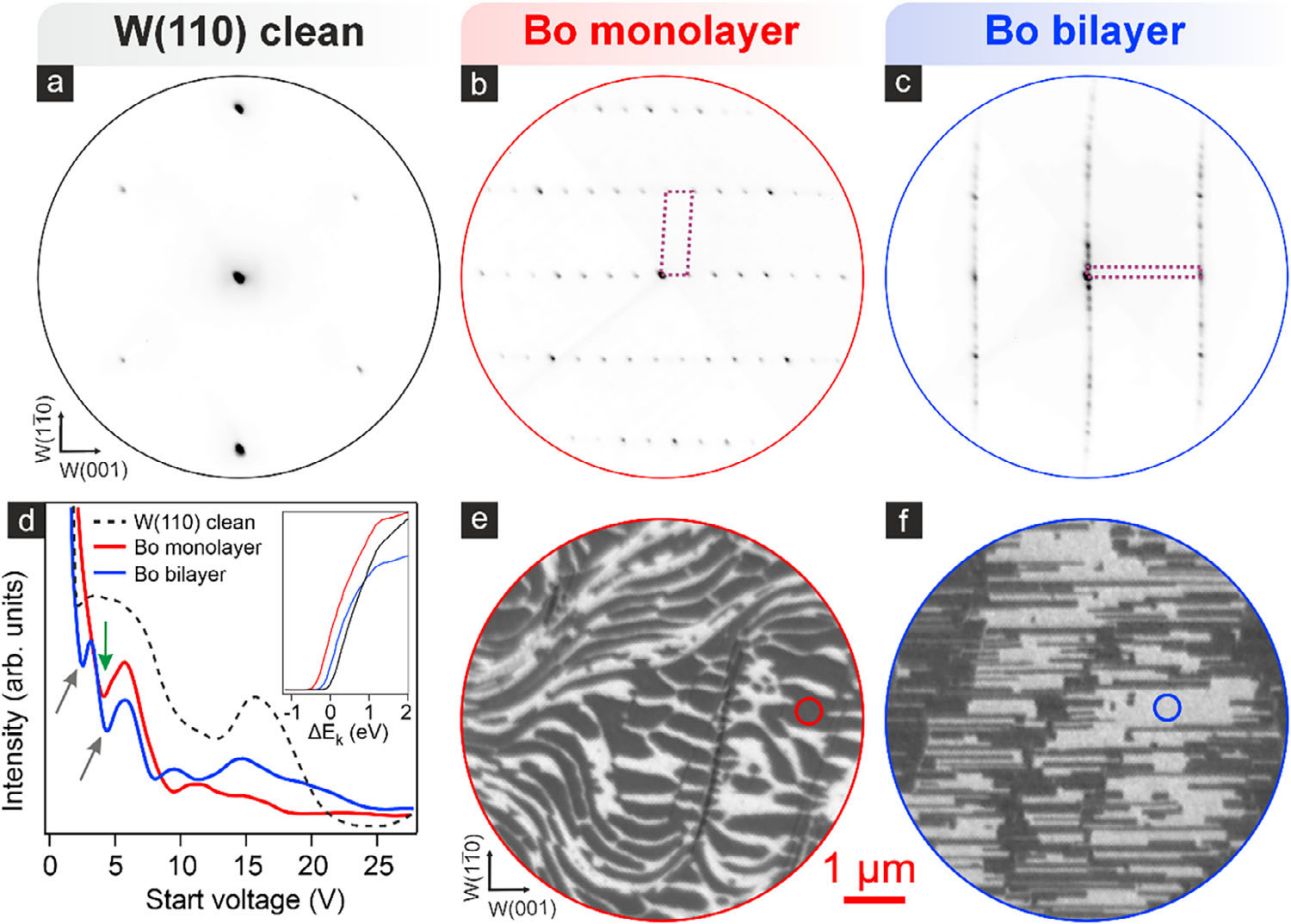
Structural characterization of monolayer and bilayer borophene on W(110). LEED patterns of: (a) clean W(110), (b) monolayer borophene/W(110) and (c) bilayer borophene/W(110). The images were acquired at an electron energy of 45 eV. (d) LEEM‐IV reflectivity curves showing one pronounced minimum for the monolayer (green arrow) and two for the bilayer (grey arrows), along with, in dashed lines, the curve of clean W(110). The inset shows the secondary‐electron cutoff spectra for the three structures in panel (d). The full spectra are shown in the Supporting Information. ΔE_k_ is defined with respect to clean W(110). LEEM images of (e) monolayer borophene (STV = 2 V) and (f) bilayer borophene (STV = 9 V).

The formation of borophene on W(110) is first investigated using LEED and LEEM to monitor the surface structural evolution during growth and annealing. Figure 1a shows the LEED pattern of the clean W(110) substrate, displaying the characteristic (1 × 1) rectangular symmetry with sharp diffraction spots, thus confirming the high crystallographic order and cleanliness of the starting surface before boron deposition.

When boron is deposited at room temperature and the sample is subsequently flash‐annealed to 1430 K, a well‐defined single‐layer boron phase is formed, characterized by a LEED pattern showing additional fractional‐order spots (Figure 1b). The ordered structure corresponds to a (1 × 9) superstructure with respect to the W(110) substrate, yielding a supercell of 4.48 × 14.42 Å^2^, enclosing an angle of 81.06° (as indicated by the purple rhombus in Figure 1b). This periodic superstructure reflects the epitaxial registry of boron with the underlying tungsten lattice and the stabilization of an ordered boron network. The monolayer phase nucleates and orders upon high‐temperature annealing, transforming an initially disordered adlayer into a uniform layer exhibiting long‐range periodicity (the evolution of the LEED pattern during growth is shown in Figure S1). The LEED pattern observed here is in agreement with previous reports on borophene formation on W(110), where the overlayer was proposed to adopt an χ_6_‐type polymorph [28].

Subsequent deposition of boron onto the monolayer phase, while maintaining the substrate at 1273 K, leads to the formation of a second borophene phase, later shown to be consistent with a bilayer structure (the evolution of the LEED pattern during growth is shown in Figure S2). The LEED pattern of this bilayer structure (Figure 1c) displays a dense array of diffraction spots along the $\left[1\bar{1}0\right]$ high‐symmetry direction of the tungsten substrate. From the diffraction pattern, a (16 × 1) supercell can be inferred, with the unit cell vectors of 35.84 × 3.17 Å^2^, enclosing an angle of 90°. The sharpness of the diffraction spots attests to the high crystalline quality of this structure. Interestingly, the LEED patterns obtained during bilayer synthesis exhibit a distinct feature: upon increasing boron deposition on top of the monolayer structure, the intensity of the diffraction spots of the (1 × 9) superstructure of the monolayer steadily decreases, while at the same time, the spots of the (16 × 1) bilayer structure appear with increasing intensity as the bilayer coverage increases. This behavior suggests that the second boron phase does not merely screen the diffraction from the underlying monolayer. Instead, the monolayer‐related LEED spots progressively lose intensity and vanish at full bilayer coverage, consistent with the disappearance of the original monolayer periodicity and the formation of a distinct ordered bilayer phase. Independent real‐space verification of this phase was reported by Hossain [29], whose STM measurements directly resolved second‐layer islands with an apparent height of 2.1 ± 0.2 Å relative to the first boron sheet. Importantly, the bilayer LEED pattern reported in that study is identical to the (16 × 1) diffraction fingerprint observed here, establishing that the two investigations probe the same long‐range‐ordered surface phase. The STM measurements, therefore, provide independent support for the bilayer assignment of the phase investigated in the present work.

The analysis of low‐energy electron microscopy‐intensity vs. voltage (LEEM‐IV) spectra provides further support that the two previously reported structures correspond to different boron thicknesses (Figure 1d), with distinct quantized interference. The reflectivity curve of the monolayer phase exhibits a single pronounced dip (as marked by the green arrow), whereas the bilayer shows two distinct minima (as marked by the grey arrows) in the range from 0 to 6 eV electron energy [30]. The inset of Figure 1d shows the secondary‐electron cutoff spectra acquired for clean W(110), monolayer borophene, and bilayer borophene (full spectra are reported in Figure S3a). The relative cutoff positions were determined from linear fits to the leading edges. Relative to clean W(110), formation of the monolayer reduces the work function by 0.37 ± 0.04 eV. Instead, the formation of the bilayer produces an increase in the work function by 0.21 ± 0.03 eV as compared to the monolayer. This evolution indicates a modification of the surface dipole that accompanies second‐layer formation and is considered alongside the XPS and ARPES results presented in the following sections.

LEEM images provide complementary morphological insight. For partial monolayer coverage (Figure 1e), borophene islands (dark in contrast at the selected electron energy, STV = 2 V) extend along the substrate terraces, following the step morphology of the substrate, with no preferential island‐edge orientation. In contrast, the bilayer islands (Figure 1f), which appear bright at the selected energy (STV = 9 V), exhibit a rectangular shape with sharply defined edges aligned along the high‐symmetry directions of the tungsten substrate.

**FIGURE 2:**
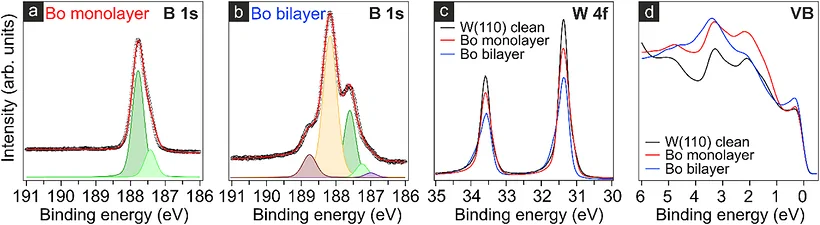
Core‐level and valence electronic structure of monolayer and bilayer borophene on W(110). X‐ray photoemission spectra at the B 1s core level and corresponding deconvolution of (a) monolayer borophene and (b) bilayer borophene. X‐ray photoemission spectra of the (c) W 4f core level and (d) valence band of clean tungsten (black), monolayer borophene (red), and bilayer borophene (blue). *hν*  =  270 eV, s‐polarization.

The chemical state of boron in the monolayer and bilayer regimes is investigated using high‐resolution XPS at the B 1s core level (Figure 2a,b). Figure 2a shows the B 1s spectrum of monolayer borophene. The spectrum is deconvoluted into two components centered at 187.43 and 187.78 eV with an integrated intensity ratio of 1:3.85. The ratio and energy position of the two peaks closely match the DFT‐simulated B 1s photoemission spectrum obtained for the freestanding χ_6_ lattice [23]. Following this theoretical model, the lower‐binding‐energy component (187.43 eV) corresponds to 4‐coordinated (B_4C_) boron atoms, while the higher‐binding‐energy peak (187.78 eV) is assigned to 5‐coordinated (B_5C_) boron atoms [31]. In contrast to the case of borophene on Ir(111) [23], where deviations between experimental and freestanding DFT‐calculated B 1s spectra indicate a significant role of substrate interaction, the present system shows a much closer agreement. In this case, the close match with the DFT simulations suggests a lower degree of substrate interaction than on Ir(111). Upon bilayer formation (Figure 2b), the B 1s line shape evolves substantially. The two monolayer‐related components shift together by approximately 0.19 eV toward lower binding energy and decrease in intensity, consistent with attenuation by the upper boron sheet. These components remain associated with the lower sheet bound to W(110), and their common shift reflects the modification of its screening upon second‐layer formation. Three additional contributions are observed at 187.03, 188.17, and 188.76 eV. The dominant component at 188.17 eV is assigned mainly to the majority boron population in the second sheet. Its higher binding energy relative to the lower‐sheet components is consistent with the weaker substrate screening experienced by the upper sheet. The weaker component at 188.76 eV is associated with a minority population with a higher coordination environment [31]. The minority‐to‐main spectral‐area ratio between the 188.76 and 188.17 eV components is 0.16. The weak component at 187.03 eV is also associated with a bilayer‐specific boron environment that may involve interlayer‐bonded boron atoms, consistent with previous DFT calculations [27]. Under identical acquisition conditions, the total B 1s area of the bilayer is 1.88 ± 0.03 times that of the saturated monolayer (comparison of B 1s photoemission spectra is found in Figure S3b).

Figure 2c compares the W 4f core‐level region acquired from clean W(110), monolayer borophene/W(110), and bilayer borophene/W(110). For the pristine surface, the W 4f_7/2_ and W 4f_5/2_ doublet exhibits relatively sharp components, characterized by a slightly asymmetric line shape, peaked at binding energies typical of metallic tungsten (31.3 and 33.4 eV) [32]. After monolayer formation, the integrated W 4f intensity decreases to approximately 90% of the clean‐W(110) value without discernible chemical shifts or line‐shape changes. This attenuation is consistent with inelastic damping of the W photoelectrons by the atomically thin boron overlayer. The absence of additional components argues against appreciable intermixing or boride formation at the interface [33]. After formation of the (16 × 1) phase, the W 4f intensity decreases further to approximately 78% of the clean‐substrate value, consistent with the addition of a second boron sheet.

Valence‐band spectra (Figure 2d) provide further evidence of the changes in the electronic configuration observed by XPS and work function measurements. Compared with the clean W(110) surface, both the monolayer and bilayer borophene samples exhibit additional features in the valence band. In particular, the clean substrate shows a plateau near the Fermi level, while both monolayer and bilayer borophene exhibit a pronounced peak in the same spectral region. Moreover, a distinct feature is observed near 3 eV for both borophene thicknesses. In this respect, density‐functional calculations and prior angle‐resolved photoemission studies have shown that borophene exhibits occupied σ‐type bands and surface‐derived states around 2.5–3.0 eV below the Fermi level [34, 35], along with borophene‐derived electronic states hybridized with W 5d orbitals.

To gain insight into the electronic structure of borophene on W(110), ARPES has been performed across the whole first and partial second surface Brillouin zone (BZ) in the −1 to 10 eV binding‐energy (BE) range for the clean substrate, monolayer borophene, and bilayer borophene.

**FIGURE 3:**
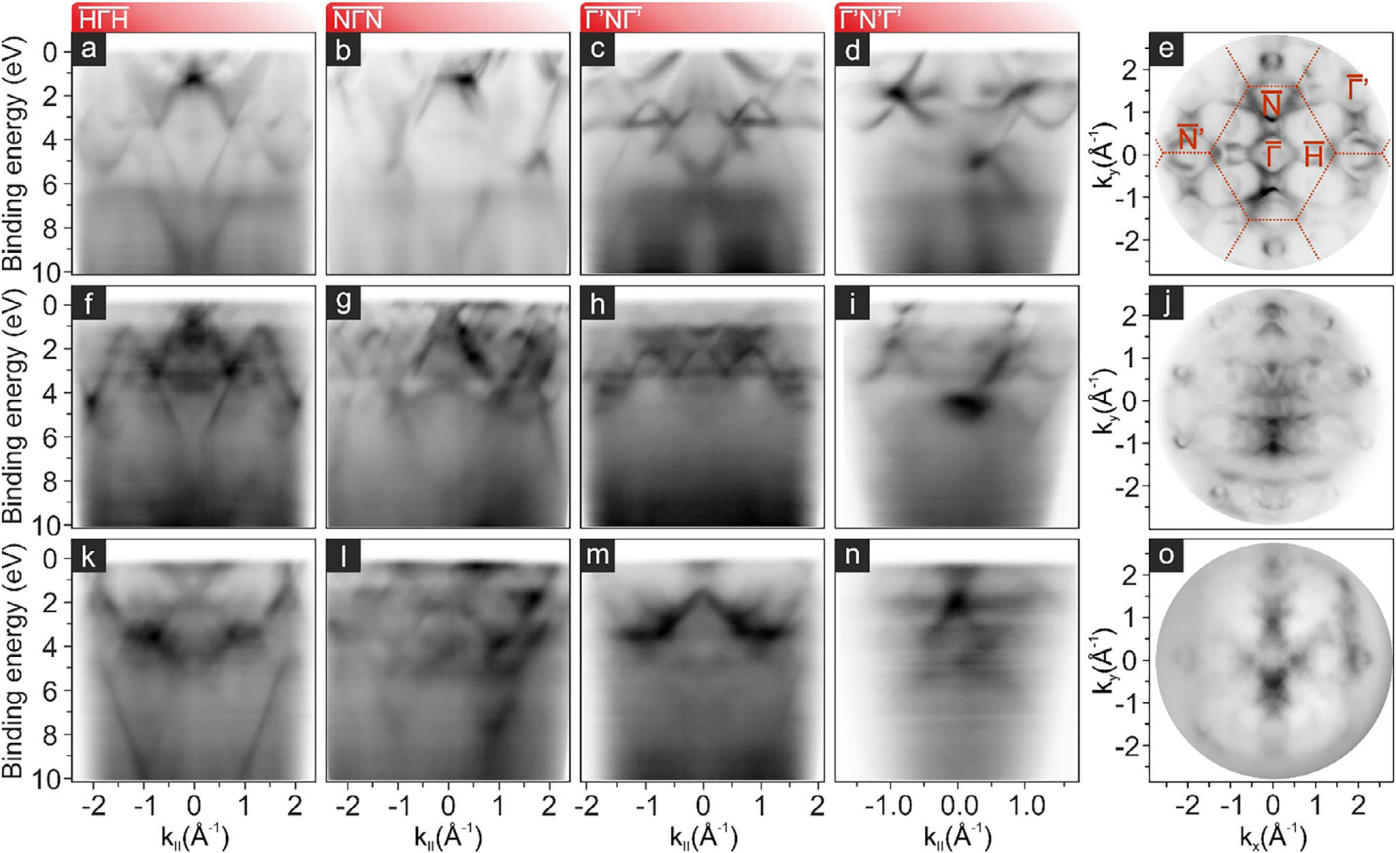
Band structure of clean W(110) and monolayer and bilayer borophene. Angle‐resolved photoemission spectroscopy of: (a–d) clean W(110), (f–i) monolayer borophene on W(110), and (k–n) bilayer borophene on W(110), acquired along the high‐symmetry directions marked on top of the figure. Corresponding Fermi surfaces of: (e) clean W(110), (j) monolayer borophene on W(110), and (o) bilayer borophene on W(110). All the spectra were acquired at room temperature with p‐polarized photons of hv = 40 eV.

The clean W(110) surface displays sharp, highly dispersive metallic bands characteristic of the tungsten 5d states (Figure 3a–d). The narrow linewidths and well‐defined crossings, characteristic of the clean tungsten surface, are in line with previous high‐resolution ARPES studies [36]. The Fermi surface (Figure 3e) exhibits elongated contours along the $\bar{\Gamma H}$ and $\bar{\Gamma N}$ directions, arising from anisotropic d‐band dispersion and strong spin‐orbit coupling intrinsic to the W(110) surface [36].

After the formation of monolayer borophene, the ARPES intensity redistributes markedly throughout the whole valence band region, reflecting both an attenuation of the tungsten photoemission signal and the appearance of new boron‐derived states (Figure 3f–i). The overall intensity in the proximity of the Fermi level increases (as also visible from Figure 2d), consistent with the partial occupation of boron π‐like states predicted for metallic borophene phases [20, 37]. Along the $\bar{H\Gamma H}$ direction, a new pair of linearly dispersing bands emerges, intersecting with the W 5d‐derived bands at approximately 1 eV binding energy to form a cone‐like dispersion. This feature is reminiscent of the band character reported for β_12_‐ and χ_3_‐type borophene sheets on Ag(111) [20, 37], while its energy position agrees with theoretical predictions for borophene phases stabilized on W(110) [34].

Along the $\bar{N\Gamma N}$ direction in the first BZ, monolayer borophene displays nearly flat bands at the Fermi level, accompanied by enhanced spectral weight around the $\bar{N}$ point and modifications of thenear‐E_F_​ states around $\bar{\Gamma }$, consistent with the density‐functional‐theory calculations for borophene π‐phase on W(110) [34]. This simultaneous presence of dispersive and nearly flat bands is typical of borophene and arises from the coexistence of highly delocalized π metallic states and more localized bonding states within the same boron lattice [20, 35]. In the $\bar{\Gamma N\Gamma }$ cut acquired across the first and the second BZ (Figure 3h), there is an enhancement in the intensity of the spectrum around the cone apex (at about 1 eV binding energy) and between 1.5‐3.0 eV BE, where boron σ‐like states are hybridized with tungsten d‐bands. A $\bar{\Gamma N\Gamma }$ cut in the second BZ (Figure 3i) reveals a clear “breaking” of tungsten‐derived bands near their crossings with boron states, indicative of hybridization gaps arising from substrate‐overlayer electronic intermixing. This effect, also observed for borophene on Ag(111) [20], demonstrates that while the borophene layer remains metallic, its bands are renormalized by the underlying substrate.

The Fermi surface of the monolayer (Figure 3j) differs strikingly from that of clean W(110). The sharp contours of the tungsten bands are replaced by a pattern of diffuse, anisotropic electron pockets extending throughout the BZ. The Fermi surface also develops additional intensity in momentum‐space regions where clean W(110) shows no detectable states. Part of this intensity may arise from superstructure‐induced umklapp replicas of substrate‐derived bands. In this respect, previous studies have shown that for monolayer borophene on Ir(111), the 2D structural modulation of the overlayer produces replication of electronic bands through photoelectron scattering from the nanograting [23]. In the present case, a similar contribution cannot be excluded; however, because of the large number of overlapping spectral features, individual umklapp‐derived bands cannot be identified unambiguously. At the same time, W‐B interaction is also evident from the modification of both the shape and intensity of the W(110) states upon monolayer borophene formation. Taken together, these observations indicate a direct interaction between borophene‐derived and tungsten‐derived states in the low‐energy region, accompanied by a redistribution of spectral weight near *E_F_*.

The electronic structure changes dramatically for the bilayer phase. The ARPES maps for the bilayer borophene reveal a broad redistribution of intensity while the monolayer's cone‐like bands are no longer resolved (Figure 3k). Instead, along $\bar{H\Gamma H}$, the spectra exhibit a pronounced V‐shaped dispersion centered at $\bar{\Gamma }$ and an enhanced, diffuse intensity between 3.0 and 4.0 eV BE, consistent with the strong valence‐band contribution observed by valence band spectroscopy (Figure 2d). Along $\bar{N\Gamma N}$ (Figure 3l), intense flat states persist near the Fermi level, while the same V‐shaped band (here fainter) is again observed at $\bar{\Gamma }$. Additionally, weaker features appear between 5‐10 eV BE only after the formation of the (16 × 1) bilayer phase, identifying them as bilayer‐related spectral weight associated with the electronic reorganization accompanying second‐layer formation and interlayer interactions.

In the $\bar{\Gamma N\Gamma }$ cut across the first and the second BZ (Figure 3m), the bilayer exhibits a cone‐like band centered at $\bar{N}$, crossing near 2.0 eV BE, accompanied by parabolic dispersions extending to larger k_∥_. Additionally, the hybridization gaps associated with the B‐W interaction in the monolayer are no longer resolved in the bilayer spectra (Figure 3n), while a similar cone‐like dispersion is clearly visible at the $\bar{N}$ point of the second BZ. For the bilayer, the Fermi surface appears largely diffuse (Figure 3o), lacking sharp contours, indicating strong broadening of the near‐Fermi‐level spectral features. These changes establish a substantial thickness‐induced reorganization of the electronic structure upon formation of the second layer. The work function increase and XPS intensity redistribution (Figures 1d and 2b) indicate changes in the surface dipole and local B environments upon second‐layer formation [20, 37].

Placed in the context of previous borophene spectroscopy studies, the present W(110) results provide a useful comparison with systems ranging from weakly perturbing to strongly corrugating substrates. On Ag(111), Dirac‐like dispersions in β_12_ borophene were measured by ARPES, demonstrating that substrate‐induced periodic perturbations can split and reshape the cones rather than suppress them entirely [20]. In parallel, spectroscopic studies on Ag‐supported borophene demonstrated charge transfer from the substrate and work‐function variations that preserve identifiable borophene‐derived electronic signatures [22]. By contrast, recent photoemission studies of monolayer borophene on Ir(111) found that the stronger, spatially modulated interaction with the substrate transforms the overlayer into a nanograting, making the direct observation of distinct borophene‐derived bands more difficult [23, 24]. Against this background, the present W(110) results appear particularly informative: the monolayer still shows clear borophene‐related fingerprints in both XPS and ARPES, including cone‐like and flat‐band features, but these coexist with B‐W hybridized features, indicating that substrate coupling does not fully suppress the borophene‐derived electronic fingerprints.

In the bilayer case, the monolayer‐related bands are no longer resolved, while new spectral features emerge. This trend is consistent with the bilayer‐borophene literature, which identifies strong interlayer interactions as one of the most important features that define the properties of the multilayer system [6, 26]. First‐principles calculations for bilayer borophene supported on Ag(111), Au(111), and Al(111) predict that metal‐induced hybridization is concentrated at the lower boron‐metal interface, with the bilayer as a whole retaining electronic features closer to those of the free‐standing system [27]. The reduced visibility of the monolayer‐specific B‐W hybridized features in the bilayer spectra is consistent with this picture. Importantly, this does not imply that the B‐W interaction disappears. The lower boron sheet may remain coupled to W(110), while formation of the upper sheet introduces new bilayer‐related contributions. However, differences at the detailed level are expected because W(110) presents strongly anisotropic d‐derived states and lower surface symmetry.

Nevertheless, the layer‐selective picture provides a useful framework for understanding the present experimental results. The (16 × 1) periodicity determines the translational symmetry of the bilayer phase but does not uniquely establish the registry between the two boron sheets, their crystallographic separation, or the distribution of interlayer B‐B bonds. A structure‐specific calculation would therefore require a dedicated search across several possible atomic configurations. Taken together, these results identify W(110) as a useful platform on which borophene retains experimentally accessible intrinsic fingerprints while revealing how thickness and the accompanying structural reconstruction jointly shape its electronic structure.

## 3 Conclusion

Monolayer and bilayer borophene on W(110) reveal how strongly the electronic structure of 2D boron is shaped by thickness and interface interactions. LEED identifies a (1 × 9) monolayer phase and a distinct (16 × 1) bilayer phase, while LEEM‐IV and quantitative XPS analysis jointly support the bilayer assignment. In situ XPS and ARPES demonstrate that these two structures are electronically nonequivalent. The monolayer retains interaction with the tungsten substrate, giving rise to hybridized and anisotropic states, whereas the bilayer displays broader valence‐band features and reduced visibility of the substrate fingerprints. Since layer addition is accompanied by structural reconstruction, the separate contributions of thickness, periodicity, and interlayer bonding cannot be fully decoupled. Beyond establishing W(110) as a robust template for ordered borophene growth, this work shows that the layer number provides an effective leverage for tuning the electronic landscape of 2D boron on anisotropic metal surfaces.

## 4 Experimental Section

### 4.1 Sample Preparation

W(110) has been used as the substrate for all experiments. The crystal has been cleaned in ultra‐high vacuum (UHV) by repeated cycles of oxygen exposure (*p*  =  3 × 10^−7^ mbar) at about 1500 K, followed by high‐temperature flashes to approximately 2300 K to remove residual oxygen. Surface cleanliness and order were confirmed by sharp (1 × 1) LEED patterns and by the absence of contamination‐related features in the W 4f core‐level spectra.

Boron has been deposited from an electron‐beam evaporator using high‐purity boron pellets. Monolayer borophene has been obtained by depositing boron onto W(110) held at room temperature, followed by a rapid flash to 1430 K and quenching to room temperature, while continuously monitoring the LEED pattern. Bilayer borophene has been prepared by depositing additional boron onto the monolayer while maintaining the substrate temperature at 1273 K. The structural evolution during both preparations was monitored in situ by LEED, while LEEM images were acquired only at selected preparation stages.

### 4.2 Experimental Methods

The experiments were performed at the Nanospectroscopy beamline of Elettra Sincrotrone Trieste S.C.p.A. using an Elmitec SPELEEM III microscope [38, 39]. The instrument can operate in both real‐space (LEEM) and reciprocal‐space (LEED) modes. The electron energy during the experiments has been defined by applying a voltage bias to the sample, commonly referred to as the start voltage (STV). The microscope allows imaging of the sample surface at video frame rate, enabling real‐time observation of dynamic surface processes with lateral resolution better than 10 nm. By inserting selected‐area apertures, spatially resolved measurements such as micro‐LEED and micro‐XPS can also be performed. When coupled to an X‐ray source with variable energy and polarization, the instrument enables chemical imaging in real‐space mode and band‐structure measurements (ARPES) in reciprocal‐space mode. The sample temperature has been measured using a type C thermocouple, directly attached to the back of the sample, with an estimated overall uncertainty of ± 50 K.

In this work, all XPS measurements were performed using 270 eV photons with s‐polarization, while ARPES measurements were acquired with 40 eV photons with p‐polarization. The overall energy resolution is about 100 meV for XPS and about 90 meV for ARPES. The binding‐energy scale has been referenced to the metallic Fermi level. Work‐function variations were determined from the secondary‐electron cutoff. Spectral deconvolution has been carried out using Voigt line shapes after Shirley background subtraction. The band structure maps were obtained from cross‐sectional cuts in the 3D‐ARPES dataset (k_x_, k_y_, E_k_), after correction of the non‐isochromaticity of the instrument [40]. Imperfections in the energy alignment, typically less than 0.05 eV, can be noted at the Fermi level (not perfectly straight). The secondary‐electron cutoff spectra were acquired in photoemission mode using hν = 270 eV and in normal emission geometry, with a sample bias STV = 2 V applied. A constant pre‐edge background was determined, and the leading edge of each cutoff was fitted with a straight line and extrapolated to the point where it intersects the background. The uncertainty was estimated by varying the fitting interval and accounting for the 0.05 eV energy step. Relative work‐function changes are reported because all spectra were acquired using identical photon energy, geometry, analyzer settings, and energy calibration.

## Conflicts of Interest

The authors declare no conflicts of interest.

## Author Contributions

**Matteo Jugovac**: conceptualization, investigation, writing – original draft, methodology, visualization, writing – review and editing, formal analysis, data curation. **Marcin Szpytma**: investigation, writing – review and editing. **Tevfik Onur Menteş**: investigation, writing – review and editing, software, resources. **Andrea Locatelli**: investigation, methodology, validation, software, resources, funding acquisition, writing – review and editing. **Iulia Cojocariu**: conceptualization, investigation, writing – original draft, writing – review and editing, validation, supervision, project administration.

## Supporting information




**Supporting File**: advs77716‐sup‐0001‐SuppMat.docx.

## Data Availability

The data that support the findings of this study are available from the corresponding author upon reasonable request.

## References

[1] A. J. Mannix , X.‐F. Zhou , B. Kiraly , et al., “Synthesis of Borophenes: Anisotropic, Two‐Dimensional Boron Polymorphs,” Science 350, no. 6267 (2015): 1513–1516, 10.1126/science.aad1080. 26680195 PMC4922135

[2] B. Feng , J. Zhang , Q. Zhong , et al., “Experimental Realization of Two‐Dimensional Boron Sheets,” Nature Chemistry 8, no. 6 (2016): 563–568, 10.1038/nchem.2491. 27219700

[3] Z. Xie , X. Meng , X. Li , et al., “Two‐Dimensional Borophene: Properties, Fabrication, and Promising Applications,” Research 2020 (2020): 2624617, 10.34133/2020/2624617. 32607497 PMC7312787

[4] Q. Zhong , L. Kong , J. Gou , et al., “Synthesis of Borophene Nanoribbons on Ag(110) Surface,” Physical Review Materials 1, no. 2 (2017): 021001, 10.1103/PhysRevMaterials.1.021001.

[5] B. Kiraly , X. Liu , L. Wang , et al., “Borophene Synthesis on Au(111),” ACS Nano 13, no. 4 (2019): 3816–3822, 10.1021/acsnano.8b09339. 30844248

[6] C. Chen , H. Lv , P. Zhang , et al., “Synthesis of Bilayer Borophene,” Nature Chemistry 14, no. 1 (2022): 25–31, 10.1038/s41557-021-00813-z. 34764470

[7] R. Wu , I. K. Drozdov , S. Eltinge , et al., “Large‐Area Single‐Crystal Sheets of Borophene on Cu(111) Surfaces,” Nature Nanotechnology 14, no. 1 (2019): 44–49, 10.1038/s41565-018-0317-6. 30510278

[8] P. Sutter and E. Sutter , “Large‐Scale Layer‐by‐Layer Synthesis of Borophene on Ru(0001),” Chemistry of Materials 33, no. 22 (2021): 8838–8843, 10.1021/acs.chemmater.1c03061.

[9] N. A. Vinogradov , A. Lyalin , T. Taketsugu , A. S. Vinogradov , and A. Preobrajenski , “Single‐Phase Borophene on Ir (111): Formation, Structure, and Decoupling from the Support,” ACS Nano 13 (2019): 14511. 31790188 10.1021/acsnano.9b08296

[10] X. Liu , Z. Zhang , L. Wang , B. I. Yakobson , and M. C. Hersam , “Intermixing and Periodic Self‐Assembly of Borophene Line Defects,” Nature Materials 17, no. 9 (2018): 783–788, 10.1038/s41563-018-0134-1. 30013053

[11] Z. Zhang , A. J. Mannix , X. Liu , et al., “Near‐Equilibrium Growth from Borophene Edges on Silver,” Science Advances 5 (2019): aax0246. 10.1126/sciadv.aax0246 PMC6764835 31598552

[12] X. Liu and M. C. Hersam , “Borophene‐Graphene Heterostructures,” Science Advances 5, no. 10 (2019): aax6444, 10.1126/sciadv.aax6444. PMC6788864 31646179

[13] K. M. Omambac , M. Petrović , P. Bampoulis , et al., “Segregation‐Enhanced Epitaxy of Borophene on Ir(111) by Thermal Decomposition of Borazine,” ACS Nano 15, no. 4 (2021): 7421–7429, 10.1021/acsnano.1c00819. 33759515

[14] S. Sheng , J.‐B. Wu , X. Cong , et al., “Raman Spectroscopy of Two‐Dimensional Borophene Sheets,” ACS Nano 13 (2019): 4133. 30913391 10.1021/acsnano.8b08909

[15] Y. V. Kaneti , D. P. Benu , X. Xu , B. Yuliarto , Y. Yamauchi , and D. Golberg , “Borophene: Two‐Dimensional Boron Monolayer: Synthesis, Properties, and Potential Applications,” Chemical Reviews 122, no. 1 (2022): 1000–1051, 10.1021/acs.chemrev.1c00233. 34730341

[16] G. Tai , T. Hu , Y. Zhou , et al., “Synthesis of Atomically Thin Boron Films on Copper Foils,” Angewandte Chemie International Edition 54, no. 51 (2015): 15473–15477, 10.1002/anie.201509285. 26510179

[17] C. Hou , G. Tai , J. Hao , L. Sheng , B. Liu , and Z. Wu , “Ultrastable Crystalline Semiconducting Hydrogenated Borophene,” Angewandte Chemie International Edition 59, no. 27 (2020): 10819–10825, 10.1002/anie.202001045. 32243024

[18] Z. Wu , X. Liang , Y. Liu , M. Xu , R. Zhu , and G. Tai , “Synthesis and Anisotropic Memristive Behavior of Borophene Nanosheets,” Angewandte Chemie International Edition 64, no. 4 (2025): 202416041, 10.1002/anie.202416041. 39223089

[19] N. R. Glavin , R. Rao , V. Varshney , et al., “Emerging Applications of Elemental 2D Materials,” Advanced Materials 32, no. 7 (2020): 1904302, 10.1002/adma.201904302. 31667920

[20] B. Feng , O. Sugino , R.‐Y. Liu , et al., “Dirac Fermions in Borophene,” Physical Review Letters 118, no. 9 (2017): 096401, 10.1103/PhysRevLett.118.096401. 28306312

[21] M. Ezawa , “Triplet Fermions and Dirac Fermions In Borophene,” Physical Review B 96, no. 3 (2017): 035425, 10.1103/PhysRevB.96.035425.

[22] X. Liu , L. Wang , B. I. Yakobson , and M. C. Hersam , “Nanoscale Probing of Image‐Potential States and Electron Transfer Doping in Borophene Polymorphs,” Nano Letters 21, no. 2 (2021): 1169–1174, 10.1021/acs.nanolett.0c04869. 33455160

[23] S. Kamal , I. Seo , P. Bampoulis , et al., “Unidirectional Nano‐Modulated Binding and Electron Scattering in Epitaxial Borophene,” ACS Applied Material Interfaces 15 (2023): 57890–57900. 10.1021/acsami.3c14884 38041641

[24] M. Jugovac , I. Cojocariu , C. A. Brondin , et al., “Coupling Borophene to Graphene in Air‐Stable Heterostructures,” Advanced Electronic Materials 9, no. 8 (2023): 2300136, 10.1002/aelm.202300136.

[25] N. Gao , X. Wu , X. Jiang , Y. Bai , and J. Zhao , “Structure and Stability of Bilayer Borophene: The Roles of Hexagonal Holes and Interlayer Bonding,” FlatChem 7 (2018): 48–54, 10.1016/j.flatc.2017.08.008.

[26] H. Zhong , K. Huang , G. Yu , and S. Yuan , “Electronic And Mechanical Properties of Few‐Layer Borophene,” Physical Review B 98, no. 5 (2018): 054104, 10.1103/PhysRevB.98.054104.

[27] W. L. Scopel , F. Crasto De Lima , P. H. Souza , J. E. Padilha , and R. H. Miwa , “Bridging Borophene and Metal Surfaces: Structural, Electronic, and Electron Transport Properties,” The Journal of Physical Chemistry C 127, no. 35 (2023): 17556–17566, 10.1021/acs.jpcc.3c03123.

[28] S. Hossain , F. J. Tuli , P. Guansong , T. Nakagawa , and S. Mizuno , “Surface Relaxation of W(110) and 2D Growth of B Studied by Low Energy Electron Diffraction and Auger Electron Spectroscopy,” Proceedings of International Exchange and Innovation Conference on Engineering & Sciences (IEICES) 6 (2020): 7–13, 10.5109/4102456.

[29] S. Hossain , Synthesis of Boron Nanostructures on Refractory Metal Surfaces (Kyushu University, 2022).

[30] H. Hibino , H. Kageshima , F. Maeda , M. Nagase , Y. Kobayashi , and H. Yamaguchi , “Microscopic Thickness Determination of Thin Graphite Films Formed on SiC From Quantized Oscillation in Reflectivity of Low‐Energy Electrons,” Physical Review B 77, no. 7 (2008): 075413, 10.1103/PhysRevB.77.075413.

[31] L. Bignardi , M. Pozzo , A. Zelenika , et al., “Determining the Atomic Coordination Number in the Structure of β12 Borophene on Ag(111) via X‐Ray Photoelectron Diffraction Analysis,” Surfaces and Interfaces 51 (2024): 104791, 10.1016/j.surfin.2024.104791.

[32] D. M. Riffe and G. K. Wertheim , “Submonolayer Oxidation of W(110): A High‐Resolution Core‐Level Photoemission Study,” Surface Science 399, no. 2‐3 (1998): 248–263, 10.1016/S0039-6028(97)00824-8.

[33] L. Yang , K. Zhang , Y. Zeng , et al., “Boron Doped *bcc*‐W Films: Achieving Excellent Mechanical Properties and Tribological Performance by Regulating Substrate Bias Voltage,” Applied Surface Science 423 (2017): 275–282, 10.1016/j.apsusc.2017.06.185.

[34] Z.‐H. Cui , E. Jimenez‐Izal , and A. N. Alexandrova , “Prediction of Two‐Dimensional Phase of Boron With Anisotropic Electric Conductivity,” The Journal of Physical Chemistry Letters 8, no. 6 (2017): 1224–1228, 10.1021/acs.jpclett.7b00275. 28247758

[35] V. V. Kulish , “Surface Reactivity and Vacancy Defects in Single‐Layer Borophene Polymorphs,” Physical Chemistry Chemical Physics 19, no. 18 (2017): 11273–11281, 10.1039/C7CP00637C. 28417128

[36] E. Rotenberg and S. D. Kevan , “Evolution of Fermi Level Crossings Versus H Coverage on W(110),” Physical Review Letters 80, no. 13 (1998): 2905–2908, 10.1103/PhysRevLett.80.2905.

[37] S. Gupta , A. Kutana , and B. I. Yakobson , “Dirac Cones and Nodal Line in Borophene,” The Journal of Physical Chemistry Letters 9, no. 11 (2018): 2757–2762, 10.1021/acs.jpclett.8b00640. 29741094

[38] A. Locatelli and E. Bauer , “Recent Advances in Chemical and Magnetic Imaging of Surfaces and Interfaces by XPEEM,” Journal of Physics: Condensed Matter 20 (2008): 093002.

[39] A. Locatelli , L. Aballe , T. O. Mentes , M. Kiskinova , and E. Bauer , “Photoemission Electron Microscopy With Chemical Sensitivity: SPELEEM Methods and Applications,” Surface and Interface Analysis 38, no. 12‐13 (2006): 1554–1557, 10.1002/sia.2424.

[40] S. Günther , T. Kratky , J. Kraus , et al., “Versatile Procedure for the Correction of Non‐Isochromatism in XPEEM Spectroscopic Imaging,” Ultramicroscopy 250 (2023): 113756. 37182363 10.1016/j.ultramic.2023.113756

