# Supplementary material for: Engineering Borophene Band Structure Through Thickness
Source: Adv Sci (Weinh). 2026 Sep 16:e77716. Online ahead of print. doi: 10.1002/advs.77716 (PMC13583102; doi:10.1002/advs.77716)
Supplement: Supplementary file 1 [file ADVS-9999-e77716-s001.docx]

Supporting Information

Engineering borophene band structure through thickness

Matteo Jugovac*, Marcin Szpytma, Tevfik Onur Menteş, Andrea Locatelli, Iulia Cojocariu*

**Figure S1. LEED image sequence acquired upon growth of monolayer borophene on W(110).** Starting from a clean tungsten surface (top left), a monolayer of boron is deposited (top middle), followed by annealing at increasing temperatures (as depicted above each panel). Finally, the monolayer borophene pattern is obtained after cooling to room temperature (bottom right). Electron energy 40 eV.

**Figure S2. LEED image sequence acquired upon growth of bilayer borophene on W(110).** Starting from a monolayer $\chi_{6}$ structure, the sequence depicts the LEED pattern upon increasing the boron evaporation amount. Electron energy 40 eV.

**Figure S3.** **Secondary electron cutoff spectra and comparison of B 1s photoemission spectra.** Secondary electron cutoff spectra for clean W(110), monolayer borophene/W(110) and bilayer borophene/W(110), acquired using STV = 2 V (a). Taking 4.68 eV as a measured value of clean W(110) work function, monolayer borophene has a work function of 4.31 eV, while bilayer has 4.51 eV. Comparison of B 1s photoemission spectra of monolayer and bilayer borophene, hν = 270 eV (b).
